# The Role of Pyroptosis and Autophagy in the Nervous System

**DOI:** 10.1007/s12035-023-03614-2

**Published:** 2023-09-12

**Authors:** Huijie Zhao, Xiaodi Fu, Yanting Zhang, Chaoran Chen, Honggang Wang

**Affiliations:** 1 Institute of Chronic Disease Risks Assessment, Henan University, Jinming Avenue, Kaifeng, 475004 China; 2 School of Basic Medical Sciences, Henan University, Kaifeng, 475004 Henan China; 3 School of Clinical Medicine, Henan University, Kaifeng, 475004 Henan China; 4 Institute of Nursing and Health, School of Nursing and Health, Henan University, Jinming Avenue, Kaifeng, 475004 China

**Keywords:** Pyroptosis, Autophagy, Nervous system, Traumatically injured spinal cord, NLRP3 inflammasome

## Abstract

Autophagy is a conservative self-degradation system, which includes the two major processes of enveloping abnormal proteins, organelles and other macromolecules, and transferring them into lysosomes for the subsequent degradation. It holds the stability of the intracellular environment under stress. So far, three types of autophagy have been found: microautophagy, chaperone-mediated autophagy and macroautophagy. Many diseases have the pathological process of autophagy dysfunction, such as nervous system diseases. Pyroptosis is one kind of programmed cell death mediated by gasdermin (GSDM). In this process of pyroptosis, the activated caspase-3, caspase-4/5/11, or caspase-1 cleaves GSDM into the N-terminal pore-forming domain (PFD). The oligomer of PFD combines with the cell membrane to form membrane holes, thus leading to pyroptosis. Pyroptosis plays a key role in multiple tissues and organs. Many studies have revealed that autophagy and pyroptosis participate in the nervous system, but the mechanisms need to be fully clarified. Here, we focused on the recent articles on the role and mechanism of pyroptosis and autophagy in the pathological processes of the nervous system.

## Introduction

### Overview of autophagy

**Fig. 1:**
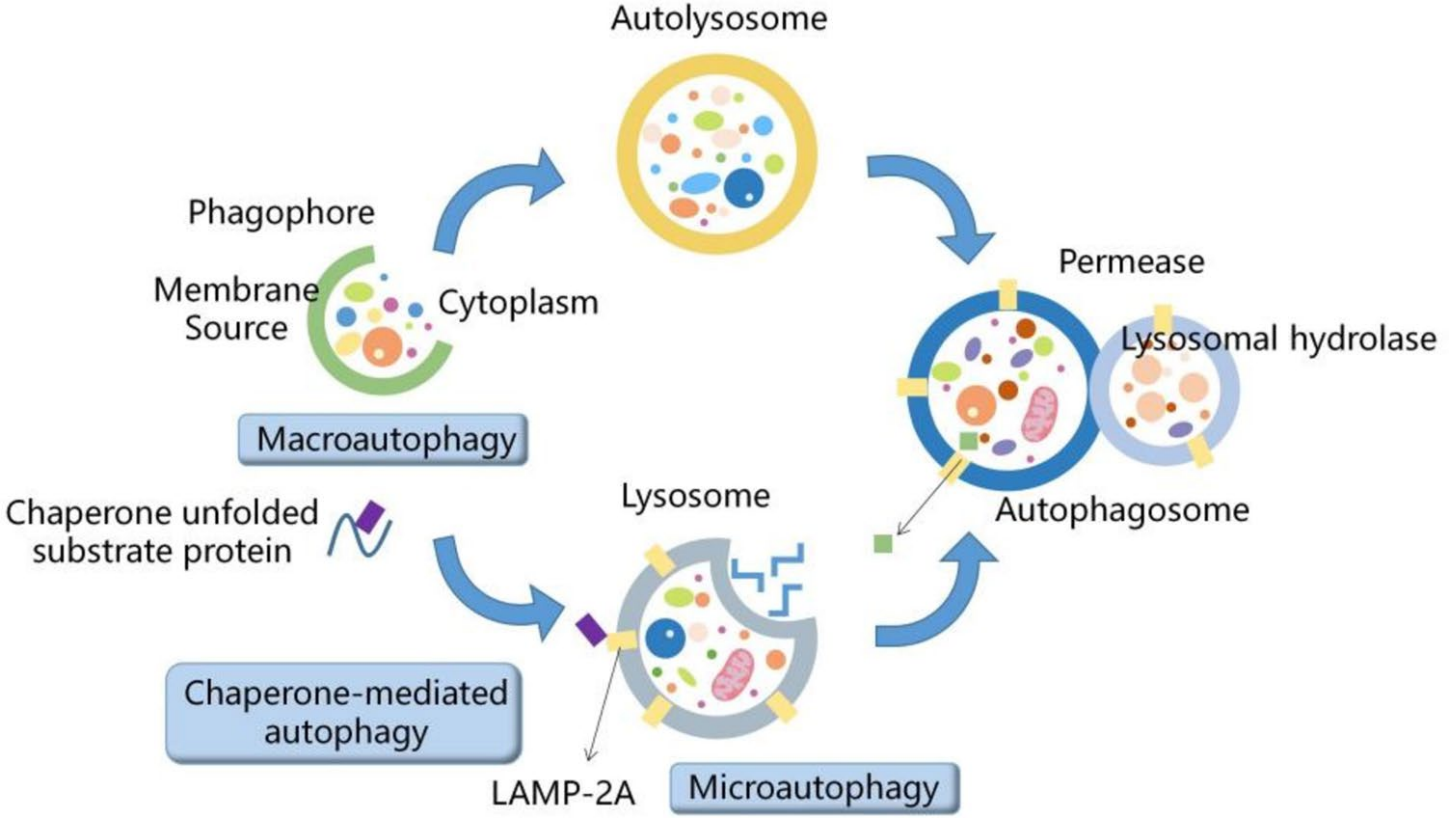
Diagram of three types of autophagy processes

As a conservative intracellular process, autophagy wraps abnormal proteins, organelles and other macromolecules, and then transfers them to lysosomes for the subsequent degradation [1–3]. Autophagy can be divided into three types according to the difference in the particularity of goods and the different delivery methods to lysosomes:chaperone-mediated autophagy, microautophagy and macroautophagy [4, 5]. Macroautophagy, which is commonly referred to as autophagy, and one of the most studied, is an evolutionarily conservative stress response process. During macroautophagy, after being isolated in double-membrane vesicles and transported to lysosomes for degradation, the invasive pathogens and abnormal organelles are degraded [6, 7]. In the process of microautophagy, the lysosome membrane is directly invaginated, and then the cell contents are wrapped [8–10]. Instead of capturing cargoes with vesicle intermediates, the chaperone-mediated autophagy transports the substrate binding with the chaperone protein directly to the lysosome cavity. To date, chaperone-mediated autophagy has only been described in mammalian cells (Fig. 1) [11–14]. Autophagy can be activated by a variety of stimuli, including glucose deprivation, nutritional starvation, caloric restriction, transient ischemia and reperfusion, and oxidative stress. Under physiological conditions, autophagy which is at a basic level helps to maintain the integrity of intracellular organelles [15, 16]. However, autophagy disorders have been observed under a wide range of pathological conditions, including obesity, infectious and inflammatory diseases, type 2 diabetes, neurodegenerative diseases and cancers [3, 17]. Neurons are a kind of polarized cells with a large amount of cytoplasm, and accumulation of cell waste will bring a heavy burden to these cells. Therefore, neurons are considered to be particularly vulnerable to autophagic dysfunction. Autophagy is important for maintaining neuronal homeostasis and eliminating protein aggregates, thus preventing neuronal disorder [18]. Autophagy also plays an important role in neuronal activity, plasticity, and memory [19]. Additionally, autophagy dysfunction often occurs in microglia [20] and astrocytes [21]. More and more evidence indicates that abnormal autophagy participates in many nervous system diseases such as Alzheimer’s disease [22], Huntington’s disease [23], and Parkinson’s disease [24]. Up to now, the role and mechanism of autophagy in the nervous system are not completely clarified.

### Overview of pyroptosis

**Fig. 2:**
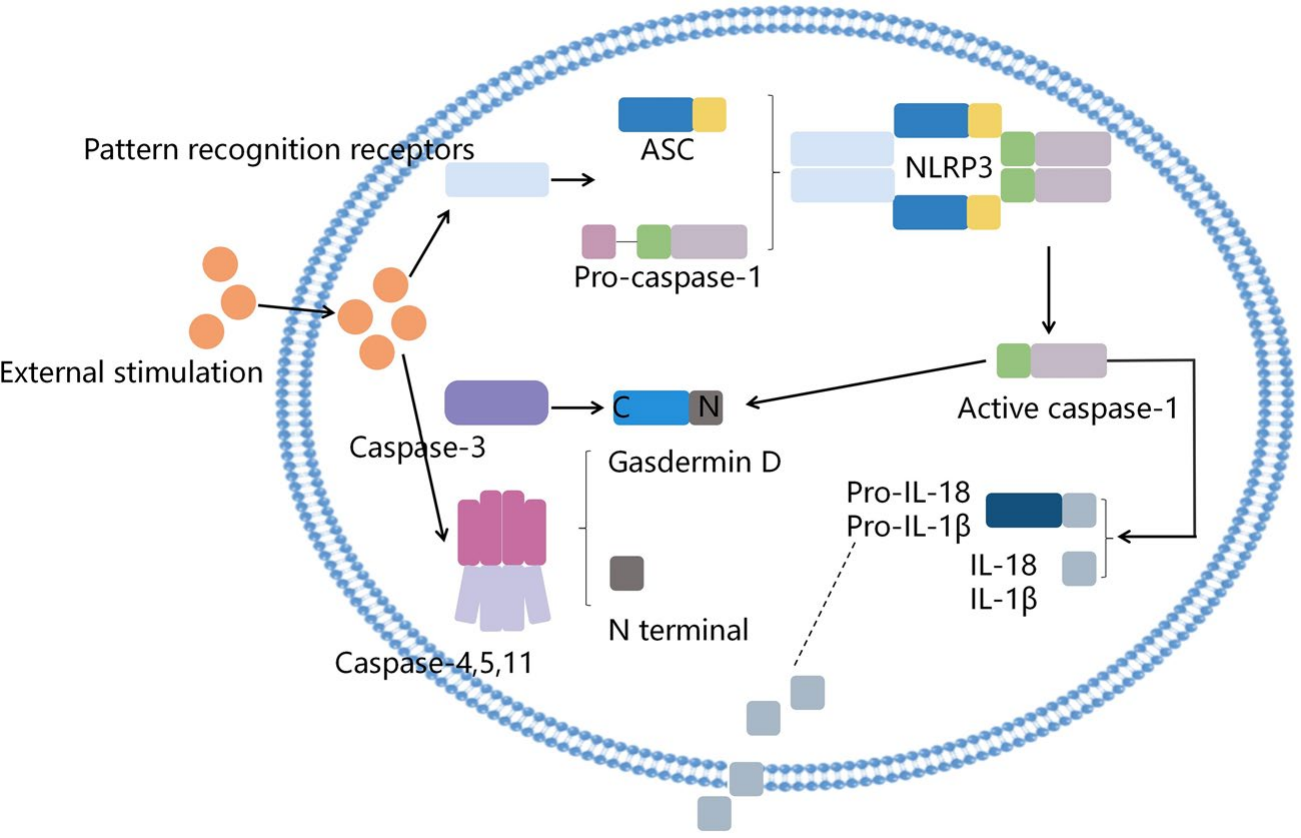
The process of three types of pyroptosis

Pyroptosis is one kind of programmed cell death mediated by gasdermin and was first discovered in Salmonella-induced macrophage death [25]. Also known as secondary necrosis, pyroptosis is a new nonapoptotic form of programmed cell death and is closely related to the inflammatory reaction [26]. It is mainly touched off by inflammasomes and is executed by the gasdermin (GSDM) family and caspases including caspase-1/- 3/- 4/- 5/- 11 [27–29]. Pyroptosis is characterized by cell swelling, the formation of large bubbles on the plasma membrane, and the rupture of the plasma membrane [30]. The GSDM family consists of six members, including DFNB and GSDM-A, -B, -C, -D, -E [31]. During pyroptosis, caspase-1 cuts GSDMD into its N-terminal fragment, which then forms cell membrane holes. Therefore, GSDMD is the main pyroptosis executor [32]. Until now, according to the different triggering modes, three kinds of pyroptosis have been found, including non-canonical pyroptosis pathway, caspase-3-mediated pyroptosis pathway and canonical pyroptosis pathway [33]. The canonical pyroptosis pathway is mediated by caspase-1 and nucleotide-binding oligomerization domain-like receptor protein 3 (NLRP3). During this process, NLRP3 inflammasome is activated, resulting in the activated caspase-1, which subsequently cuts GSDMD into its N- and C-terminal fragments. The N-terminal fragments then bind with the cell membrane to form pyroptotic holes [34]. The non-canonical pyroptosis pathway is mediated by human caspases-4 and-5 and mouse caspase-11. The caspase recruitment domain (CARD) of the gram-negative bacteria directly binds to lipopolysaccharide (LPS), triggering non-standard inflammatory pathways. Caspase-4/5/11 can directly cut GSDMD into an N-terminal fragment and a C-terminal fragment. The N-terminal domain then binds with the cell membrane to produce pyroptotic pores, which release interleukin-1beta (IL-1β) and IL-18 out of cells, thus activating the process of non-canonical pyroptosis [35]. In pyroptosis mediated by caspase-3, caspase-3 cleaves GSDME into the GSDME-N domain that binds with the cell membrane to result in pyroptotic pores, thereby triggering pyroptosis [36, 37]. See Fig. 2 for the above three pathways of pyroptosis [38, 39]. The evidence indicates that pyroptosis occurs in multiple types of nervous system cells, including microglia, astrocytes, oligodendrocytes, neurons and peripheral cells [40]. Therefore, pyroptosis is involved in many pathological processes of the nervous system, such as multiple sclerosis, Alzheimer’s disease, stroke, traumatic brain injury (TBI) and spinal cord injury (SCI), resulting in neuroinflammation and neurodegeneration through a variety of mechanisms. The treatment of targeted pyroptosis has shown promise in various preclinical models of nerve injury and diseases [41, 42]. Recently, the evidence indicates that autophagy and pyroptosis are involved in the nervous system, but the mechanisms have not been completely clarified. Hence, we focus on the recent progress in the role and mechanisms of pyroptosis and autophagy in the pathological processes of the nervous system.

## The role of pyroptosis and autophagy in microglia

### The role of pyroptosis and autophagy in microglia with hypoxemia

The typical symptom of acute respiratory distress syndrome (ARDS) is persistent hypoxemia, which often requires ventilation to correct hypoxemia. The ventilatory strategies can lead to hypercapnia [43, 44]. It has been reported that in hypoxic adult rats, hypercapnia can induce IL-1β overproduction by activating nucleotide-binding oligomerization domain-like receptor protein 3 (NLRP3) inflammasome [45, 46]. However, its mechanism is not completely clear. In addition, mitophagy plays a vital role in the ischemic model of Alzheimer’s disease [47–49]. To seek the role and mechanism of hypercapnia and mitophagy in ARDS, Hong guang Ding and colleagues conducted a lot of experiments, and the results showed that hypercapnia reduced the partial pressure of oxygen (PbtO2) levels in the brain tissue of hypoxic rats, and this was further demonstrated by an increase of cerebral oxygen extraction ratio (CERO2) levels. Hypercapnia decreased the expression of microtubule-associated protein light chain-II (LC3-II) and upregulated p62 expression in hypoxic microglia, indicating that hypercapnia downregulated microglia mitophagy. The in-depth research showed that hypercapnia upregulated mitochondrial reactive oxygen species (ROS) production, and activated NLRP3 inflammasome, thus promoting microglia pyroptosis, as evidenced by the increased expression of GSDMD-N in hypoxia-activated microglia, while the scavenging of ROS could suppress microglial pyroptosis and decrease the expressions of IL-18 and IL-1β [50]. Evidence indicates that mitophagy inhibits NLRP3 inflammasome activation by clearing mitochondrial ROS [51, 52]. Therefore, it can be inferred from the above that hypercapnia promotes NLRP3 inflammasome-mediated microglial pyroptosis by suppressing mitophagy via increasing mitochondrial ROS overproduction in hypoxemic adult rats [50]. Parkin RBR E3 ubiquitin-protein ligase (PARKIN)/PTEN-induced kinase 1 (PINK1) pathway has been reported to regulate mitophagy in nervous system diseases [53, 54]. Hence, whether hypercapnia inhibited mitophagy through PINK1/PARKIN remains to be studied.

### The role of pyroptosis and autophagy in microglia with pneumococcal infection

The gram-positive bacterium *Streptococcus pneumoniae* can cause pneumonia, otitis media, meningitis and other diseases with the characteristics of high mortality and high incidence rate [55–57]. However, the exact mechanism of its pathogenesis is not completely clarified. Ji Yun Kim and colleagues demonstrated that *S. pneumoniae* promoted pyroptosis by inducing caspase-1 activation and IL-1β production in murine microglia. NLRP3 siRNA treatment reduced the levels of IL-1β IL-18 and caspase-1 in murine microglia infected by *S. pneumoniae.* NLRP3 siRNA also decreased lactate dehydrogenase (LDH) release of *S. pneumoniae*-infected murine microglial cells. These indicated that NLRP3 inflammasome mediated *S. pneumoniae*-promoted pyroptosis. Moreover, *S. pneumoniae* infection upregulated autophagy by increasing the expressions of autophagy-related genes and promoting autophagosome formation in the early stage of pneumococcal infection (4 h). The inhibition of autophagy upregulated the expressions of IL-18, caspase-1 and IL-1β in *S. pneumoniae*-infected murine microglia, and induced the release of LDH, IL-1β and IL-18 of *S. pneumoniae*-infected murine microglia, indicating that autophagy suppressed *S. pneumoniae*-induced NLRP3 caspase-1/inflammasome-induced pyroptosis. These suggested that the promotion of autophagy by *S. pneumoniae* transiently inhibited *S. pneumoniae*-induced pyroptosis in microglia in the early stage of pneumococcal infection. Furthermore, in murine microglia, *S. pneumoniae* increased ROS production, while NAC(a ROS scavenger) downregulated the *S. pneumoniae*-induced ROS production, and inhibited autophagy, suggesting that *S. pneumoniae* promoted autophagy via increasing ROS. Moreover, pneumococcal infection-induced ROS suppressed the activation of caspase-1 within 4 h after infection. But in the late stage of infection, ROS induced caspase-1-dependent microglia pyroptosis and IL-1β secretion. From the above, it can be deduced that *S. pneumoniae*-induced ROS upregulated autophagy to inhibit microglia pyroptosis in the early stage of infection (within 4 h). However, *S. pneumoniae*-induced ROS promoted pyroptosis in the late phase of infection [58]. The increased ROS from mitochondria or other intracellular compartments promotes pyroptosis and autophagy [59]. Therefore, it can be inferred as follows: in the early stage of *Streptococcus pneumoniae* infection, the increased ROS can activate autophagy to suppress NLRP3 inflammasome-mediated microglia pyroptosis, while the enhanced autophagy can clear ROS. With the extension of infection time, ROS is cleared by autophagy and reduced to a certain extent, and its tendency to activate autophagy greatly decreases, while its role of activating NLRP3 inflammasome-mediated microglia pyroptosis appears, which shows that *S. pneumoniae* induced microglia pyroptosis [58].

Nucleotide-binding oligomerization domain 2(NOD2), a member of the leucine-rich repeat receptor (NLR) family, can detect the specific bacterial peptidoglycans and regulate the expressions of inflammatory factors by activating the nuclear transcription factor-kappa B (NF-kB) pathway [60]. Receptor-interacting protein 2 kinase (RIP2) is activated by intracellular NOD2 and is involved in bacterial infection-induced inflammatory response [61]. It has been reported that the NOD2-RIP2 pathway participates in *S. pneumoniae* infection [62], but the relevant mechanisms still require to be fully clarified. The studies of Guan Wang et al. revealed that *S. pneumoniae* upregulated the level of microglial ROS, LDH release and the secretion of IL-18 and IL-1β, strengthened caspase-1 activity, and decreased microglial viability, indicating that *S. pneumoniae* promoted microglial ROS production and pyroptosis. Furthermore, *S. pneumoniae* upregulated the levels of autophagy, NOD2, RIP2 and phospho-ULK1 (p-ULK1, an important regulator of autophagy) in microglial. Treatment of microglia with 6-(tert-Butylsulfonyl)-N-(5-fluoro-1H-indazol-3-yl)quinolin-4-amine (GSK583, a RIP2 kinase inhibitor), as well as knockdown of NOD2 or RIP2, decreased the levels of p-ULK1 and autophagy-related proteins, suggesting that NOD2-RIP2 pathway promoted microglia autophagy via phosphorylating ULK1. In *Streptococcus pneumoniae*-infected microglia, the knockdown of ULK1 increased the ROS production, the secretion of IL-1β, IL-18 and LDH release and caspase-1 activity, and reduced the levels of microglia viability and autophagy-related proteins, indicating that ULK1-regulated autophagy inhibited microglia ROS production and pyroptosis. Similar to the results in vitro, the NOD2-RIP2 pathway promoted autophagy in the brain of the *Streptococcus pneumoniae*-infected mouse. Furthermore, the inhibition of ULK1 or RIP2 notably promoted microglia pyroptosis of the brain in *Streptococcus pneumoniae*-infected mice. Collectively, the NOD2-RIP2 pathway inhibited microglia pyroptosis by promoting ULK1-regulated autophagy during the infection of *Streptococcus pneumoniae* [63]. Evidence indicates that ROS can activate NLRP3 inflammasome that in turn promotes NLRP3 inflammasome-mediated pyroptosis [64]. In the above study, although NLRP3 inflammasome level was not detected, from the result that the NOD2-RIP2 pathway inhibits ROS generation, it can be deduced that autophagy suppresses NLRP3 inflammasome-induced pyroptosis via reducing ROS, which needs further confirmation.

### The role of pyroptosis and autophagy in microglia with oxygen–glucose deprivation/reoxygenation

Neonatal hypoxic–ischemic brain damage (HIBD) is the principal reason for diseases, including epilepsy, cerebral palsy and cognitive impairment in children [65, 66]. To date, there is still no effective treatment for HIBD, which has a serious impact on children’s health and life quality. There is an urgent need to seek effective treatment for HIBD [67, 68]. It has been reported that mesenchymal stem-cell-derived exosomes (MSC-exos) can ameliorate neonatal HIBD [69]. But the relevant mechanism needs to be completely clarified. Zhenzhen Hu and colleagues successfully collected MSC-exos from human MSC, as evidenced by identifying the diameter, the shape and the marker protein of exosomes. MSC-exos promoted microglia viability, reduced the levels of caspase-1, NLRP3 and GSDMD-N, and the release of IL-18 and IL-1β in microglia induced by OGD/R, suggesting that MSC-exos suppressed OGD/R-induced microglia pyroptosis. Compared with the medium from OGD/R-exposed and PBS-treated microglia, the medium from OGD/R-exposed and MSC-exos-treated microglia notably upregulated microglia viability and decreased LDH release, suggesting that MSC-exos mitigated OGD/R-induced microglia damage. MSC-exos also increased the levels of COX IV and TOM20, two mitophagy-related proteins, in microglia induced by OGD/R. Meanwhile, mitochondrial division inhibitor-1 and 3-methyladenine(3-MA) alleviated MSC-exos suppression of pyroptosis, suggesting that MSC-exos suppressed OGD/R-induced microglia pyroptosis through upregulating mitophagy. Additionally, MSC-exos notably upregulated the expression of Forkhead box 3a(FOXO3a) in OGD/R-exposed microglia. The siRNA of FOXO3a partially abolished the neuroprotection of MSC-exos and alleviated MSC-exos-mediated suppression of pyroptosis and promotion of mitophagy, suggesting that MSC-exos promoted mitophagy to suppress OGD/R-induced microglia pyroptosis by promoting FOXO3a. Summarily, MSC-exos mitigated ischemia/reperfusion(I/R)-induced microglia pyroptosis by upregulating the expression of FOXO3a to promote mitophagy [70]. Mitophagy maintains intracellular environment stability through selectively eliminating the injured mitochondria [71]. Therefore, mitophagy suppresses NLRP3 inflammasome activation through decreasing the mitochondrial ROS that can activate NLRP3 inflammasome [72]. It can be inferred that mitophagy suppresses microglia pyroptosis via inhibiting NLRP3 inflammasome in microglia with oxygen–glucose deprivation/reoxygenation.

### The role of pyroptosis and autophagy in microglia with inflammation

Dimethyl itaconate (DI), a cell-permeable derivative of the endogenous metabolite itaconate, has been regarded as an anti-inflammatory regulator of macrophages [73, 74]. But the effect of DI on inflammasome-mediated pyroptosis remains unknown. Su Yang and colleagues found that lipopolysaccharide (LPS) + ATP promoted NLRP3 inflammasome activation, LDH release and GSDMD cleavage in microglia, which was reversed by DI or NLRP3 siRNA, indicating that DI inhibited NLRP3-dependent microglia pyroptosis [75]. The evidence showed that M1-polarized microglia can generate pro-inflammatory cytokines to promote inflammation. Conversely, M2-polarized microglia can generate anti-inflammatory cytokines to inhibit inflammation [76, 77]. LPS + ATP induced the transition of microglia from M2 polarization to M1 polarization, increased the levels of inflammatory mediators and activated the NF-κB pathway, which was reversed by DI, indicating that DI suppressed LPS + ATP-induced microglia inflammation. Moreover, DI induced the activation of heme oxygenase‑1 (HO‑1)/nuclear factor erythroid 2‑related factor 2 (Nrf‑2) pathway, and the inhibition of Nrf-2/HO-1 pathway abolished DI protection of microglia, suggesting that DI inhibited NLRP3-dependent microglia pyroptosis by activating Nrf-2/HO-1 pathway. Meanwhile, DI also reduced LPS + ATP-induced ROS production in microglia. In addition, DI promoted LPS + ATP-inhibited autophagy, while suppression of autophagy with 3-MA mitigated DI effects on the levels of IL-1β, cleaved GSDMD and NLRP3, indicating that DI played an anti-inflammatory role by inducing autophagy. Collectively, DI inhibited LPS + ATP-promoted and NLRP3 inflammasome-mediated microglia pyroptosis by promoting Nrf-2/HO-1 pathway and autophagy [75]. Evidence indicates that ROS promotes NLRP3 inflammasome-mediated pyroptosis [78, 79]. Autophagy suppresses NLRP3 inflammasome via clearing mitochondrial ROS and degrading NLRP3 inflammasome components [13]. Therefore, autophagy inhibited NLRP3 inflammasome-mediated pyroptosis by clearing ROS and degrading NLRP3 inflammasome components in microglia in the above study [75].

## The role of pyroptosis and autophagy in diabetes nervous system diseases

### The role of pyroptosis and autophagy in diabetes-induced brain injury

Diabetes is a vital risk factor for ischemic stroke. The prognosis of diabetes patients after ischemic stroke is poor [80, 81]. The evidence from animal models of diabetes shows that neuronal death is a vital factor leading to brain injury [82, 83], and caspase-1 inhibition mitigates brain ischemic injury-induced neuronal death [84, 85]. Hui Che and colleagues found that melatonin treatment notably inhibited neuronal death in both streptozotocin (STZ)-treated diabetic mice and high glucose (HG)-induced neuronal cells. Furthermore, melatonin suppressed neuronal pyroptosis and enhanced autophagy induced by diabetes through reducing the levels of Beclin1, NLRP3, ATG12, caspase-1, GSDMD-N, and LC3 in vivo and in vitro. The in-depth research revealed that MicroRNA-214-3p (miR-214-3p) level was downregulated in HG-induced neuronal cells and diabetic mice, which was reversed by melatonin. MiR-214-3p overexpression decreased the levels of IL-1β, caspase-1 and GSDMD-N, while the inhibition of miR-214-3p had the opposite effects on HG-induced neuronal cells, indicating that miR-214-3p suppressed pyroptosis. In addition, the inhibition of miR-214-3p abolished the melatonin suppression of pyroptosis and autophagy induced by diabetes in vitro. Summarily, melatonin played a neuroprotective role via suppressing neuronal autophagy and pyroptosis induced by diabetes by promoting miR-214-3p [86]. Melatonin may be a vital candidate drug for the treatment of brain injury with diabetes. The relationship between autophagy and pyroptosis in diabetes nervous system diseases needs to be clarified.

### The role of pyroptosis and autophagy in diabetes cerebral ischaemia diseases

Hypothermia is considered one of the neuroprotective strategies, which can significantly improve cerebral ischemia injury in non-diabetic animal models [87, 88]. However, whether hypothermia can improve diabetes-aggravated cerebral ischemia injury is not completely clear. To clarify this, Yanling Tu et al. introduced permanent middle cerebral artery occlusion (pMCAO) into the rat model of type 2 diabetes that was constructed by a high-fat diet combined with intraperitoneal injection of STZ in vivo. Moreover, the HG stimulation and OGD/R were used to establish the cell model of cerebral ischemia with diabetes in vitro. The subsequent studies revealed that diabetes aggravated brain edema and cerebral infarction, and worsened the nerve defect of cerebral ischemia, which was improved by hypothermia. Diabetes increased the blood–brain barrier (BBB) permeability through upregulating matrix metalloproteinase 9 (MMP-9) expression and promoting the degradation of tight junction proteins, and hypothermia reversed this phenomenon. Diabetes upregulated the expressions of GSDMD, caspase-1, NLRP3 and p62, and downregulated microtubule-associated protein 1 light chain 3B (LC3B) II/I ratio in the rat model of cerebral I/R injury with diabetes, which were abolished by hypothermia, suggesting that hypothermia increased autophagy and downregulated pyroptosis. Furthermore, 3-MA reduced the LC3 II/I ratio and increased the levels of p62, caspase-1, NLRP3 and GSDM-N, suggesting that hypothermia inhibited pyroptosis via inducing autophagy. Baf (an autophagy inhibitor) significantly increased the levels of GSDM-N, NLRP3 and caspase-1 inhibited by hypothermia, indicating that hypothermia could suppress pyroptosis by promoting autophagy via promoting the fusion of lysosome and autophagosome. Summarily, diabetes-aggravated cerebral I/R injury was ameliorated via hypothermia by inhibiting pyroptosis and inducing autophagy, which needed to be further verified [89]. Contrary to some of the above conclusions, the upregulation of autophagy can aggravate cerebral ischemia injury [90]. The reason may be related to the duration of cerebral ischemia, which requires to be further study. The study shows that the activation of autophagy protects neurons from cerebral I/R injury by clearing damaged mitochondria [91]. Therefore, it can be deduced that autophagy can suppress cell pyroptosis via clearing mitochondrial ROS, which needs to be further verified [89].

Contrary to the above result that the activation of autophagy plays a protective role against cerebral I/R injury, the suppression of autophagy also improves cerebral I/R injury. Spautin-1 is an autophagy inhibitor and can induce the degradation of the Vps34 PI3 complex by suppressing USP10 and USP13 (two ubiquitin-specific peptidases) [92]. Hui Liu et al. established the model rats of cerebral I/R injury in vivo through middle cerebral artery occlusion for 60 min and reperfusion for 24 h, and the models of cerebral I/R injury in vitro models with PC12 cell through OGD/R. The results revealed that spautin-1 alleviated cerebral I/R injury through reducing infarct size and ameliorating cerebral I/R-induced neurological impairment in vivo. In OGD/R-induced PC12 cells, spautin-1 also increased cell viability, and decreased ROS production and the number of autophagic microsomes. The in-depth research showed that spautin-1 suppressed NLRP3 inflammasome-mediated pyroptosis and autophagy induced by cerebral I/R through downregulating the expression levels of NLRP3, Beclin 1, USP13 and GSDMD-N in vivo and in vitro. However, the overexpression of USP13 counteracted spautin-1 improvement of cerebral I/R injury and significantly abolished spautin-1 suppression of autophagy and NLRP3 inflammasome-dependent pyroptosis. Collectively, spautin-1 protected against cerebral I/R injury by suppressing autophagy/pyroptosis via the inhibition of USP13 [93]. The relationship between pyroptosis and autophagy in the above study needs to be elucidated.

## The role of pyroptosis and autophagy in traumatically injured spinal cord

Spinal cord injury (SCI) is a destructive neuropathological disease that can lead to major motor, sensory, and autonomic dysfunction [94, 95]. The evidence indicates that pyroptosis plays an important role in SCI [96–98]. Yu Xu and colleagues found that the growth differentiation factor 11 (GDF-11) significantly optimized functional recovery of SCI by decreasing the glial scars and increasing the number of SYN-positive synapses on neurons and the expression of neuronal microtubule-associated protein-2 (MAP2). GDF-11 inhibited pyroptosis by reducing the levels of pyroptosis-associated markers after SCI, including NLRP3, caspase-1, apoptosis-associated speck-like protein (ASC), IL-1β, IL-18 and GSDMD. Moreover, GDF-11 upregulated the levels of Beclin1, LC3II, CTSD (an autolysosome-related marker), and VPS34 (an autophagosomal marker) in neurons, but downregulated p62 level, indicating that GDF-11 activated autophagy. The co-treatment GDF-11 with 3-MA downregulated the levels of autophagy-related markers, and upregulated the levels of pyroptosis-associated markers after SCI, and abolished the conducive influences of GDF-11 on SCI, suggesting that autophagy mediated GDF-11 effects. Mechanism research revealed that GDF-11 upregulated TFE3 expression in neurons, while TFE3 siRNA reversed GDF-11 effects on autophagy and pyroptosis, indicating that GDF-11 promoted autophagy and suppressed pyroptosis by activating TFE3. In addition, GDF-11 activated the AMPK-TRPML1-Calcineurin pathway after SCI, while compound C (an AMPK inhibitor) inhibited the AMPK-TRPML1-Calcineurin pathway and TFE3, and abolished the above GDF-11 effects on autophagy, pyroptosis and improvements of SCI, indicating that AMPK-TRPML1-Calcineurin pathway mediated GDF-11 effects on TFE3, autophagy, pyroptosis and improvement of SCI. Summarily, GDF-11 ameliorated SCI by inhibiting neuron pyroptosis through promoting TFE3-mediated autophagy via activating the AMPK-TRPML1-Calcineurin pathway [99]. Autophagy suppresses pyroptosis by inhibiting NLRP3 inflammasome, and GDF-11-targeting autophagy/pyroptosis may be a new therapeutic method for SCI.

## Conclusion

**Fig. 3:**
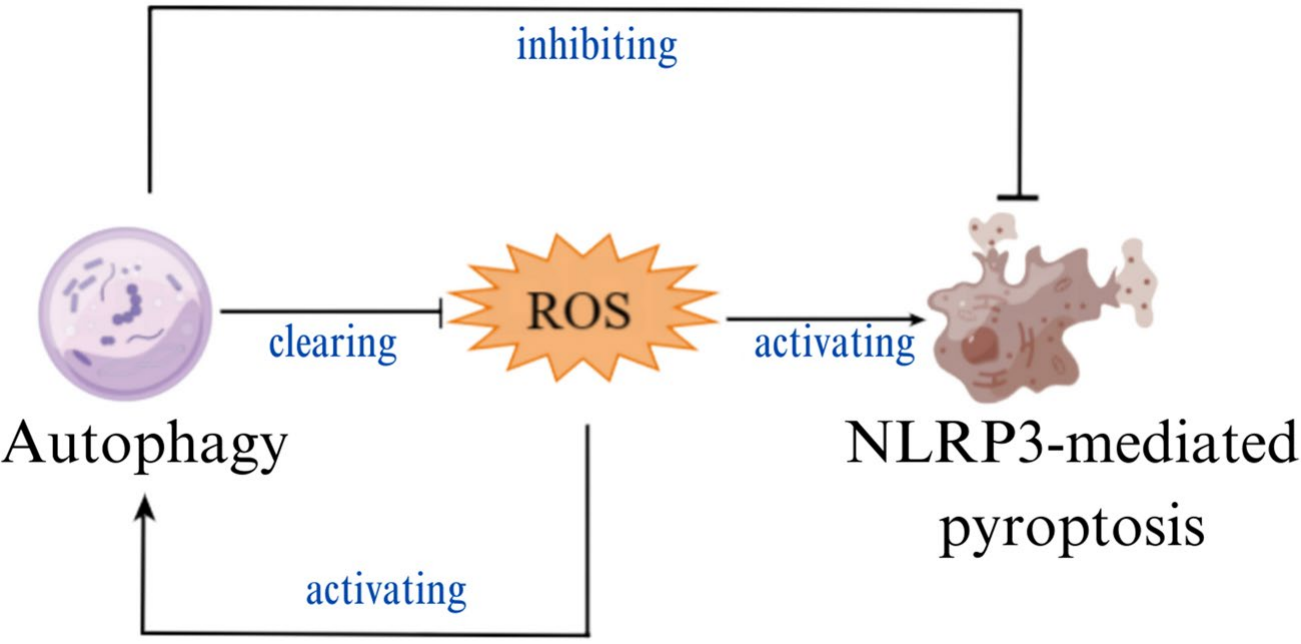
Reactive oxygen species (ROS) regulates autophagy and pyroptosis in nervous system

The increasing evidence shows that pyroptosis and autophagy play a vital role in the pathological processes of the nervous system. Here, we focused on the role of pyroptosis and autophagy in the pathological processes of the nervous system as follows: (1)hypercapnia promotes NLRP3 inflammasome-mediated microglial pyroptosis by inhibiting mitophagy through increasing mitochondrial ROS production in hypoxemic adult rats; (2)*Streptococcus pneumoniae* inhibits microglia pyroptosis by upregulating autophagy via ROS in the early stage of infection; (3)NOD2-RIP2 pathway suppresses microglial pyroptosis by promoting ULK1-regulated autophagy during streptococcus pneumonia infection; (4) MSC-exos alleviates microglial pyroptosis induced by I/R through upregulating the expression of FOXO3a to promote mitophagy; (5) dimethyl itaconate suppressed NLRP3 inflammasome-mediated and LPS + ATP-induced microglia pyroptosis via inducing autophagy and activating Nrf-2/HO-1 pathway; (6) melatonin inhibits neuronal pyroptosis and autophagy induced by diabetes through promoting miR-214-3p; (7) hypothermia improves diabetes-promoted cerebral I/R injury via inhibiting pyroptosis through inducing autophagy; (8) spautin-1 improves cerebral I/R injury via suppressing autophagy/pyroptosis through suppressing inhibition of USP13; (9) the growth differentiation factor 11(GDF-11) ameliorates SCI by inhibiting neuronal pyroptosis through promoting TFE3-mediated autophagy via activating AMPK-TRPML1-Calcineurin pathway (Table 1). It can be concluded from the above that the signaling pathways, including the NOD2-RIP2 pathway, Nrf-2/HO-1 pathway, and AMPK-TRPML1-Calcineurin pathway, are involved in the role of pyroptosis and autophagy in pathological processes of the nervous system. Whether there are other signal pathways involved in autophagy/pyroptosis in the nervous system remains to be further studied. There are two mechanisms for autophagy to negatively regulate pyroptosis: one is that autophagy suppresses pyroptosis through clearing pathogen-associated molecular patterns (PAMPs) and damaging associated molecular patterns (DAMPs). The other is that autophagy suppresses pyroptosis through suppressing pyroptosis components [100]. In this review, autophagy inhibits NLRP3 inflammasome-mediated neuronal pyroptosis by clearing mitochondrial ROS. Whether the other mechanism for autophagy to negatively regulate pyroptosis is involved in the role of pyroptosis and autophagy in pathological processes of the nervous system needs to be studied. Furthermore, in the above-cited literature, it is clarified that ROS can also activate autophagy, and regulate autophagy/pyroptosis, and ROS is an important node connecting autophagy and pyroptosis (Fig. 3 by Figdraw). How ROS regulates autophagy/pyroptosis in the nervous system requires to be further clarified. In addition to the NLRP3/caspase-1/GSDM pathway, whether autophagy can regulate neuronal pyroptosis through 3/- 4/- 5/- 11/GSDM is worthy of future research.

**Table 1:** The summary of the role of autophagy and pyroptosis in pathological processes of nervous system

The type of the pathological processes of the nervous system	The role of autophagy and pyroptosis	Experimental model	Reference
Microglia with hypoxemia	Hypercapnia promotes NLRP3 inflammasome-mediated microglial pyroptosis by inhibiting mitophagy through increasing mitochondrial ROS production	Hypoxemic adult rats	[50]
Microglia with pneumococcal infection	*Streptococcus pneumoniae* inhibits microglia pyroptosis by upregulating autophagy via ROS in the early stage of infection	Rats with pneumococcal infection	[58]
Microglia with pneumococcal infection	NOD2-RIP2 pathway suppresses microglia pyroptosis by promoting ULK1-regulated autophagy during Streptococcus pneumonia infection	Mouse meningitis model	[63]
Microglia with oxygen–glucose deprivation/reoxygenation	MSC-exos alleviates I/R-induced microglia pyroptosis via increasing FOXO3a expression to promote mitophagy	Oxygen–glucose deprivation/reperfusion (OGD/R)-induced mice microglia	[70]
Microglia with inflammation	Dimethyl itaconate suppressesNLRP3 inflammasome-mediated and LPS + ATP-induced microglia pyroptosis by promoting autophagy and activating Nrf-2/HO-1 pathway	Mice/mice microglia with inflammation	[75]
Neurological disease with diabetes	Melatonin inhibits neuronal pyroptosis and autophagy induced by diabetes through promoting miR-214-3p	Streptozotocin (STZ)-induced diabetic mice	[86]
Neurological disease with diabetes	Hypothermia improves diabetes aggravated cerebral I/R injury via inhibiting pyroptosis through inducing autophagy	STZ-treated diabetic mice and high glucose (HG)-induced neuronal cells	[89]
Neurological disease with diabetes	Spautin-1 improves cerebral I/R injury via suppressing autophagy/pyroptosis through suppressing inhibition of USP13	Rats with middle cerebral artery occlusion/PC12 cell with OGD/R	[93]
Traumatically injured spinal cord	Growth differentiation factor 11(GDF-11) ameliorated SCI by inhibiting neurons pyroptosis through promoting TFE3-mediated autophagy via activating AMPK-TRPML1-Calcineurin pathway	Mice with traumatically injured spinal cord	[99]

In this review, in most cases, autophagy and pyroptosis are mutually exclusive, but in a few cases, it is the opposite. Pyroptosis and autophagy can both repel each other and coexist in different nervous system cases. The reason may be related to different pathological processes and is required to be clarified.

It is believed that autophagy and pyroptosis will become new targets for the treatment of the nervous system.

In macroautophagy, the invasive pathogens and abnormal organelles are degraded after being isolated and transported to lysosomes in double-membrane vesicles (autophage) for degradation. In microautophagy, the direct invagination of lysosome membrane and then the encapsulation of cell contents. Companion-mediated autophagy does not capture goods with vesicular intermediates, but directly transports the substrate bound with chaperone protein to lysosomal cavity.

Autophagy inhibits ROS activation of NLRP3 inflammasome-mediated pyroptosis by clearing ROS. Autophagy also inhibits pyroptosis by inhibiting NLRP3 inflammasome. The elevated ROS activates autophagy and NLRP3 inflammasome-mediated pyroptosis.
